# Supplementary material for: A gain and dynamic range independent index to quantify spillover spread to aid panel design in flow cytometry
Source: Sci Rep. 2021 Oct 15;11:20553. doi: 10.1038/s41598-021-99831-7 (PMC8520008; doi:10.1038/s41598-021-99831-7)
Supplement: Supplementary file 1 — Supplementary Information. [file 41598_2021_99831_MOESM1_ESM.pdf]

Supplementary Information

A gain and dynamic range independent index to quantify spillover spread to aid panel design in flow cytometry

Debajit Bhowmick*[1], Frank van Diepen[2], Anita Pfauth[2], Renaud Tissier[3] , Michelle Ratliff[4]

1. Flow Cytometry Facility, Brody School of Medicine, East Carolina University

600 Moye Blvd, Greenville, NC 27858

2. Flow Cytometry Facility, Netherlands Cancer Institute – Antoni van Leeuwenhoek Hospital

Plesmanlaan 121, 1066 CX, Amsterdam, Netherlands

3. Senior Biostatistician, Netherlands Cancer Institute – Antoni van Leeuwenhoek Hospital

Plesmanlaan 121, 1066 CX, Amsterdam, Netherlands

4. Department of Microbiology and Immunology, Brody School of Medicine, East Carolina University

600 Moye Blvd, Greenville, NC 27858

Correspondence to: Debajit Bhowmick

email: bhowmikdebajit@gmail.com

Phone: 252 744 3245

Detector	B695		V450	V610	V710	Y582	Y610	Y670	Y780	R670		R730	
Fluorochrome	BB700	PerCP-Cy5.5								Alexa 647	APC	APC R700	Alexa 700
**BB700**			29	161	1864	8	17	41	72	232	341	483	1695
**PerCP-Cy5.5**			20	26	258	8	9	56	63	53	78	117	410
**Alexa Fluor 647**	69	555	21	25	48	8	10	80	51			223	782
**APC**	119	954	20	30	147	8	12	134	55			162	568
**APC-R700**	42	335	17	26	223	7	9	35	116	386	569		
**Alexa Fluor 700**	21	171	20	25	96	7	9	15	45	23	34		

Supplementary Table S1: Effect of multiple fluorochrome in one detector on SQI: In this table we have presented a special scenario, where B695, R670 and R730 are associated with two fluorochromes. Data indicates same amount of spread (as channel number) translates in bigger SQI when a dim fluorochrome is associated with the secondary detector.

Laser	BD FACSAri Fusion 1 and 3			BD FACSAri Fusion 2			BD FACSSymphony A5			ACEA NovoCyte Quanteon			BC CytoFLEX S	
	Long Pass	Band Pass	Laser Power (mW)	Long Pass	Band Pass	Laser Power (mW)	Long Pass	Band Pass	Laser Power (mW)	Mirror	Band Pass	Laser Power (mW)	Band Pass	Laser Power (mW)
				770	820/60		770	819/44						
				-	379/28		690	735/50						
							630	670/25						
**355**			NA			50	600	615/20	100			NA		NA
							550	580/20						
							450	515/30						
							-	379/28						
	750	780/60		750	780/60		750	780/60		757LP	780/60		450/45	
	690	710/50		690	710/50		735	750/30		-	725/40		525/40	
	630	660/20		630	660/20		685	711/25		705LP	695/40		610/20	
**405**	595	610/20	85	595	610/20	100	635	661/11	100	685LP	660/20	100	660/20	80
	505	525/50		505	525/50		595	605/40		598SP	615/20			
	-	450/50		-	450/50		550	586/15		570SP	586/20			
							455	470/20		552LP	530/30			
							-	431/28		495LP	445/45			
	655	695/40		655	695/40		750	780/60		757LP	780/60		690/50	
	502	530/30		502	530/30		735	750/30		-	725/40		525/40	
	-	488/10		-	488/10		685	710/50		705LP	695/40		488/10	
**488**			50			100	635	661/11	100	685LP	660/20	100		50
							600	610/20		598SP	615/20			
							570	586/15		570SP	586/20			
							505	530/30		552LP	530/30			
							-	488/10						
	755	780/60		755	780/60		750	780/60		757LP	780/60		660/20	
**640**	690	730/45	100	690	730/45	140	690	730/45	100	-	725/40	100	712/25	50
	-	670/30		-	670/30		-	670/30		705LP	695/40		780/60	
										685LP	660/20		-	
	735	780/60		735	780/60		750	780/60		757LP	780/60		585/42	
	685	710/50		685	710/50		685	710/50		-	725/40		610/20	
	630	670/14		630	670/14		635	670/30		705LP	695/40		690/50	
**561**	600	610/20	50	600	610/20	50	600	610/20	100	685LP	660/20	100	780/60	30
	-	582/15		-	586/15		-	586/15		598SP	615/20			
										570SP	586/20			
										552LP	561/14			

Supplementary Table S2: Configuration detail: This table lists the optical configurations of the instruments that were used in this study.

	Antibody	Fluorochrome	Clone	Catalog	Vendor	(ng of antibody/test)	
Panel 1	CD45	Alx488	HI30	304019	Biolegend	250	
Panel 1	CD3	BV605	UCHT1	300459	Biolegend	400	
Panel 1	CD123	BV711	6H6	306030	Biolegend	125	Panel 2
Panel 1	TCRγδ	PerCP-eFluor 710	B1.1	46-9959-42	Thermo	500	Panel 2
Panel 1	CD4	APC	SK3	566915	BD	100	Panel 2
Panel 1	CD8	BUV805	SK1	612889	BD	125	Panel 2
Panel 1	CD25	BUV395	2A3	564034	BD	500	Panel 2
Panel 1	CD127	PE	HIL-7R-M21	557938	BD	500	Panel 2
	CD3	APC-R700	UCHT1	565120	BD	400	Panel 2
	CD45	BV650	HI30	304043	Biolegend	250	Panel 2

Supplementary Table S3: Antibody details of the validation assay.

SQI		Detector					
		APC	APC-R700	Alexa Fluor 488	BV605	BV650	PerCP-eFluor 710
Fluorochrome	APC		16	11	36	58	26
	APC-R700	104		16	24	45	523
	Alexa Fluor 488	23	10		6	18	4
	BV605	66	30	17		46	27
	BV650	231	42	21	141		51
	PerCP-eFluor 710	113	176	14	36	42	

Supplementary Table S4: Use of SQI in panel design: Antibodies from Supplementary Table S3 used to saturatedly stain antibody capture beads to calculate SQI.

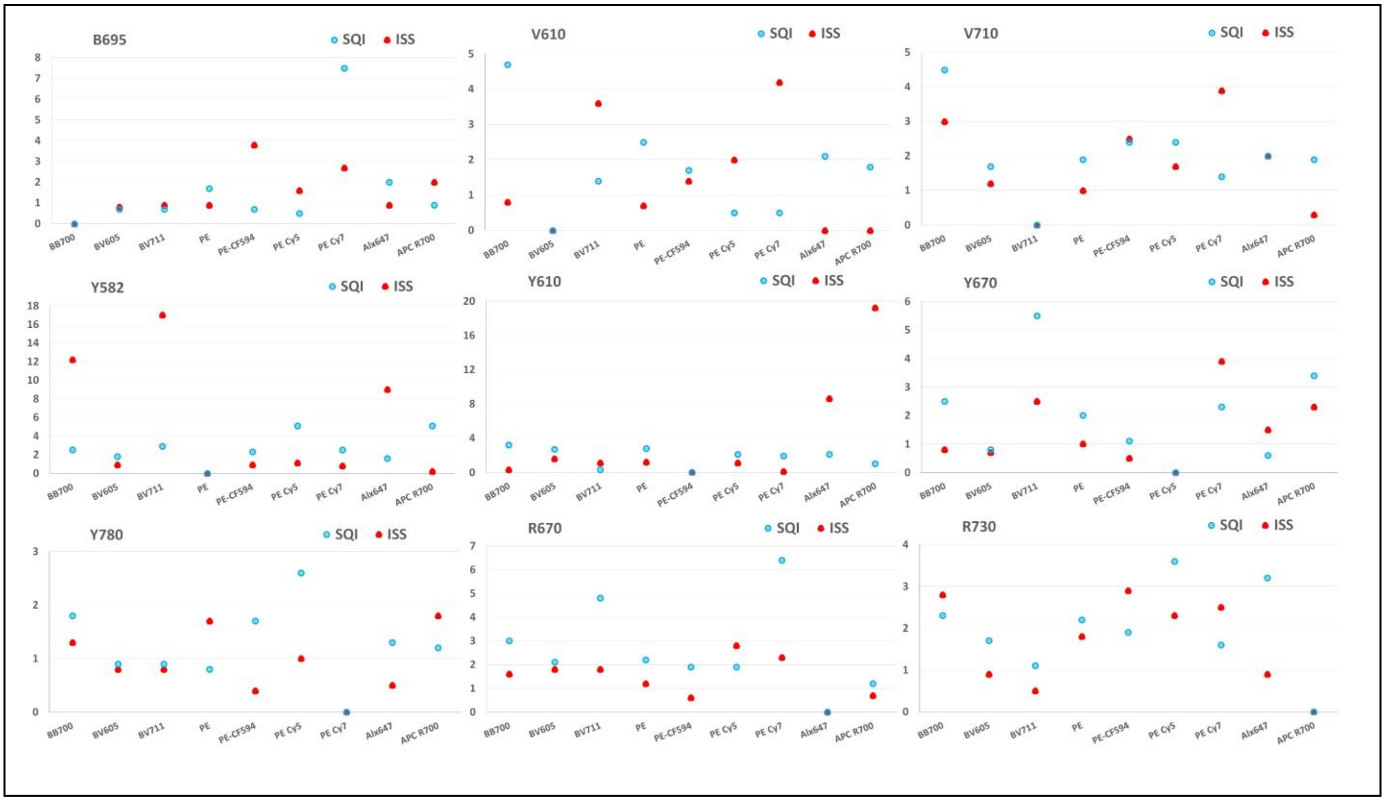

Supplementary Figure S1: Reproducibility of SQI: Three replicative data sets from Fusion 1 were used to calculate SQI and ISS values. Each dot represents the relative standard deviation (%) of three runs. Blue circle and Red triangle represent SQI and ISS respectively.

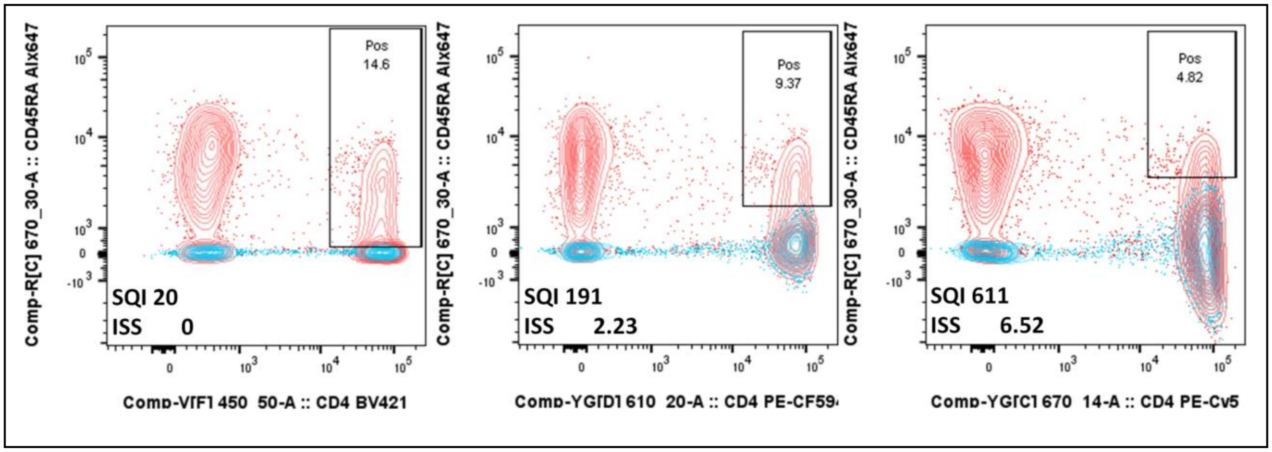

Supplementary Figure S2: Usefulness of range: Alexa Fluor 647 conjugated CD45RA was combined with CD4 BV421, PE-CF594 or PE-Cy5 (clone matched), separately. The concentration of the CD45RA antibody was equal in all mixes. The blue contour indicates the control sample, which was stained with a CD4 antibody only. The red contour shows the combined staining. SQI and ISS values are displayed. The double positive population is better separated from the single positive population at lower SQI values as is exhibited by a higher percentage.

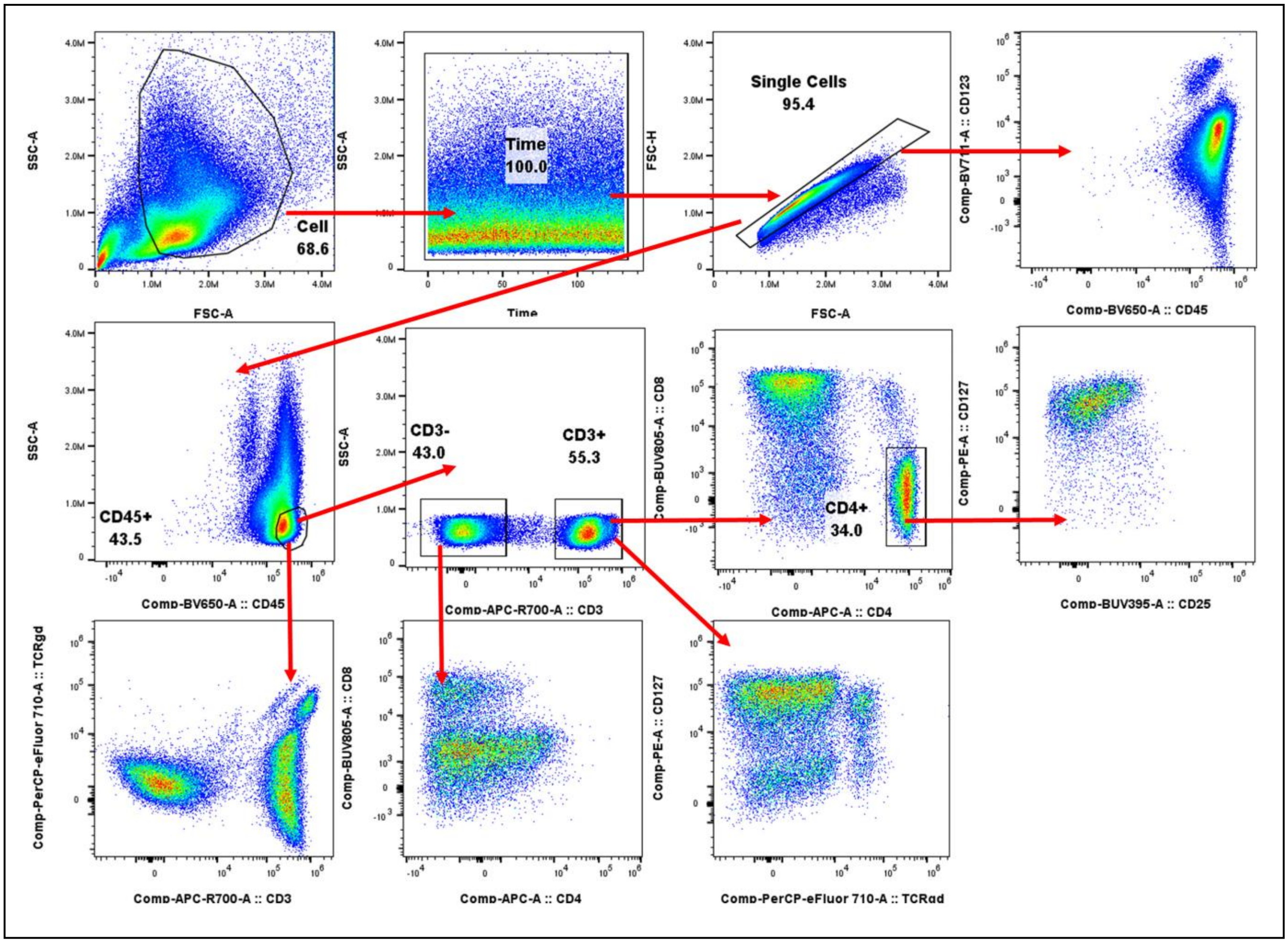

Supplementary Figure S3: Analysis hierarchy of the validation assay. PBMCs were stained for flow cytometric analyses. Data were analyzed for the populations indicated. Data was acquired on a Cytek Aurora (full spectral cytometer).

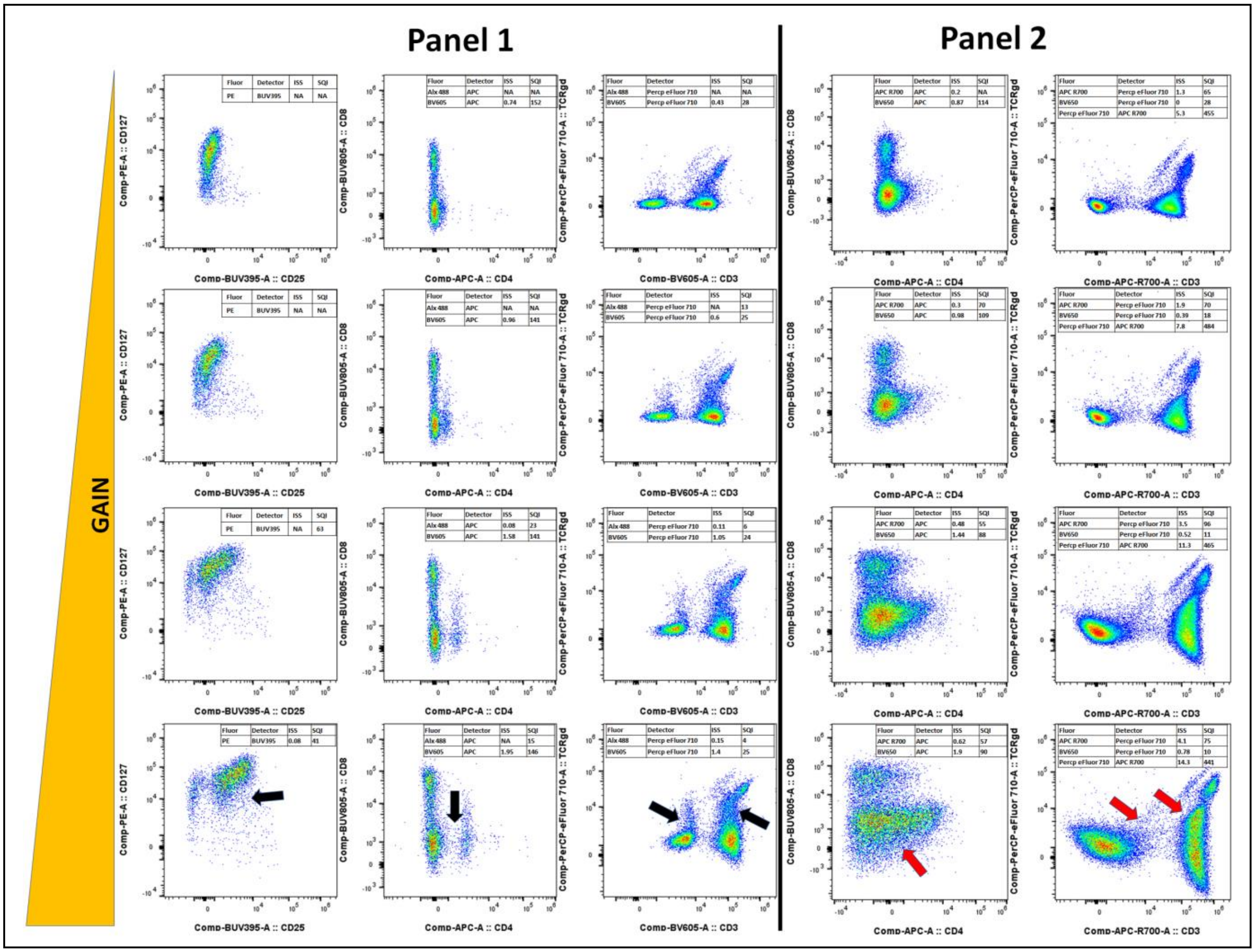

Supplementary Figure S4: Changes to detector sensitivity artificially alters data presentation. Specific populations of PBMCs stained as in S4 are shown. Data was acquired on a Cytek Aurora (full spectral cytometer), with standard gain values used (Cytek Assay Settings, CAS; 3rd row across), with additional acquisition runs with gain in all channels increased by 80% (bottom row across) and gain in all channels decreased by 50% (2nd row across) and 70% (Top row across). Panel 1 and Panel 2 differ in 2 fluorochrome labels, CD3-BV605, CD45-Alexa Fluor 488 and CD3-APC-R700, CD45-BV650, respectively. Black arrows indicate populations that lose resolution as the gain is decreased, and Red arrows indicate populations masked by spread and decreased gain. Numbers in each plot indicate the corresponding ISS and SQI values, where N/A is used when the MFI of the populations are out of the linear range of the detector.
